# Optimizing Type 1 Diabetes Screening in People With Family History: A German Perspective

**DOI:** 10.1177/19322968251383911

**Published:** 2025-11-10

**Authors:** Thomas Danne, Thomas M. Kapellen, Sebastian A. Widholz, Martin Wabitsch, Ralph Ziegler

**Affiliations:** 1 NOVA Medical School, Lisbon, Portugal; 2 Breakthrough T1D (formerly known as JDRF), New York, NY, USA; 3 Hospital for Children and Adolescents, University of Leipzig, Leipzig, Germany; 4 Sciarc GmbH, Baierbrunn, Germany; 5 Division of Pediatric Endocrinology and Diabetes, Department of Pediatrics and Adolescent Medicine, University of Ulm, Ulm, Germany; 6 German Center for Child and Adolescent Health (DZKJ), Partner Site Ulm, Ulm, Germany; 7 Diabetes Clinic for Children and Adolescents, Muenster, Germany

**Keywords:** antibody screening, autoimmune disease, diabetes, familial autoimmune disease, first-degree relatives, German perspective, screening, type 1 diabetes

## Abstract

Individuals with a family history of type 1 diabetes (T1D) are at significantly higher risk of developing T1D compared to the general population. Before its clinical onset, individuals with T1D can be identified through islet autoantibody (IAb) testing which, if multiple IAbs are detected, justifies the diagnosis of early-stage T1D. Amid rising global T1D incidence, we outline Germany’s strategy for early detection and management focused on individuals with a family history and, where informative, implementation lessons are illustrated using findings from the German Fr1da general-population study. Genetic risk factors for T1D development in individuals with family history are discussed, as well as impacts of positive screening results including influence on diabetic ketoacidosis (DKA) rates and psychological aspects. In parallel, recommendations and consensus guidelines from other national screening efforts are introduced. Building on this, we address challenges in nationwide T1D family-based screening integration and explore leveraging health care systems for cost-effective implementation. We also provide practical aspects to overcome barriers for family-based T1D screening and introduce monitoring strategies in individuals with early-stage T1D. With the advent of disease-modifying therapies (DMTs) for delaying T1D progression, there is now a rationale at hand that offers an IAb screening incentive. Collectively, we emphasize the critical role of early detection and monitoring among at-risk relatives in mitigating the burden of T1D on individuals, families, and health care systems.

## Introduction

Presymptomatic type 1 diabetes (T1D) arises when autoreactive T lymphocytes destroy beta cells, causing systemic insulin deficiency. This autoimmune process is associated with the occurrence of islet autoantibodies (IAbs) that are directed against beta cell antigens. Typically, IAbs can be detected years before the clinical onset of T1D in the blood of affected individuals, allowing a staging system for T1D disease: In stage 1, serological autoimmunity against two or more antigens is present, accompanied by normoglycemia. This is followed by stage 2, in which dysglycemia is experienced, and stage 3, at which point typical symptoms (such as polyuria, polydipsia, weight loss or fatigue) occur due to persistent hyperglycemia.^1^ Around 85% of children with multiple IAbs against different beta cell antigens develop a clinically overt T1D within 15 years after the timepoint of seroconversion. The lifetime risk for clinical onset of T1D in individuals with ≥2 IAbs approaches 100%.^2^ Once manifested, the long-term nature of T1D leads to persistent perturbation of glucose homeostasis, for example, elevated levels of glycated hemoglobin HbA_1c_. An often life-threatening acute complication of uncontrolled hyperglycaemia is diabetic ketoacidosis (DKA). Furthermore, metabolic dysregulation and associated long-term outcomes such as microvascular or macrovascular complications increases the risk of individuals with T1D for experiencing cardiovascular disease.^3^

Although incidence peaks at 10 to 14 years and is rising 3% to 4% per year,^4^ 62% of new diagnoses worldwide occur in people ≥20 years.^5^ In Germany, adults comprised 91% of the estimated 372 000 people living with T1D in 2024, and incidence has increased by 2.4% over recent years, with a notable surge during the COVID-19 pandemic, reaching 29.9 per 100 000 person-years in 2020;^6^ males remain more frequently affected than females (31.9 vs 26.5).^7^ In other countries, similar rising trends are observed.^8^ By 2040, an estimated 1.5 million adolescents under 20 worldwide will be living with T1D,^5^ driving up health care costs and necessitating enhanced management strategies to meet this growing challenge for care providers and health systems both in Germany and worldwide.^9^

**Figure 1.**
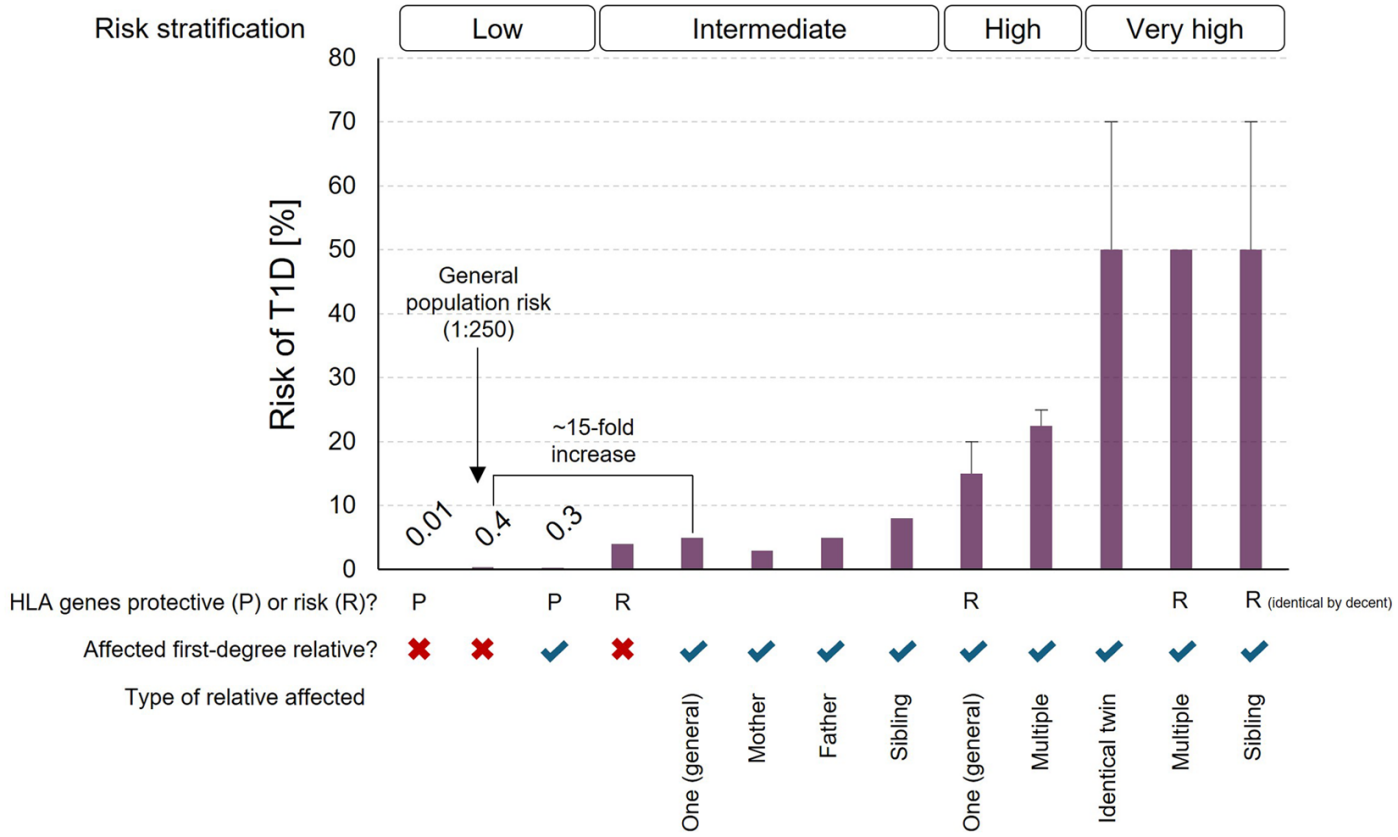
Lifetime risk of developing T1D according to genetic status (protective or risk HLA genotypes) and family history of T1D. Data from Ziegler and Nepom.^24^

Several environmental and genetic factors shape T1D risk, with the strongest heritable component residing in human leucocyte antigen (HLA) class II alleles (variants DR3-DQ2 and DR4-DQ8 account for 40%-50% of genetic predisposition and appear in up to 90% of people with T1D).^10^ Beyond HLA genes, at least 78 loci including *INS* and *PTPN22* polymorphisms contribute to autoimmunity,^11-13^ and when combined into a polygenic genetic risk score (GRS) can predict islet autoimmunity and T1D onset, although genetic influence diminishes with age.^14,15^ In the general population, lifetime T1D incidence is about 1:250,^16^ but first-degree relatives face markedly higher risks (Figure 1, 1:40 for children of affected mothers, 1:15 for children of affected fathers, and 1:35-1:12 for siblings with an estimate of roughly 10% of these relatives developing T1D by age 60).^17,18^ Monozygotic twins show 30% to 70% concordance, underscoring environmental contributions even within shared genetics,^19^ yet 90% of cases arise without any family history.^20^ Clinically, relatives diagnosed through early screening present younger, with lower DKA rates and HbA_1c_ at onset, though postonset progression mirrors that of nonfamilial cases.^21^ Relatives thus represent a vital target for presymptomatic detection and intervention, including disease-modifying therapies (DMTs) to preserve beta cell function, and for empowering families to prepare psychologically and practically for life with T1D. This is associated with preserving life expectancy, preventing neurocognitive decline and easing the financial and psychological burdens on families.^22^ Early-stage diagnosis is furthermore linked to reduced hyperglycemia severity, fewer DKA events, higher residual C-peptide and smoother transitions to insulin therapy with improved adherence.^23^

To our knowledge, no overview on family-history-based German T1D screening has been published. We therefore review ongoing screening strategies for their potential implication in the German health care system.

## Current Landscape of Screening Efforts

### The German Perspective

Stage 1 T1D is established by detecting two or more IAbs—insulin autoantibodies (IAAs), glutamate decarboxylase antibodies (GADA), tyrosine phosphatase antibodies (IA2A), zinc transporter-8 antibodies (ZnT8A), and cytoplasmatic islet cell antibodies (ICA)—with progression risk determined by age at seroconversion, antibody titer, and the number of distinct IAbs.^1,25^ Seroconversion peaks around 1 year of age, predominantly IAAs, whereas GADAs emerge later.^26^ Building on the predictive power of IAb screening, attention has increasingly shifted toward screening first-degree relatives and those with a family history of T1D. In Germany, the guideline of the German Diabetes Society (DDG) states that through determination of IAbs and follow-up, children and adolescents in stages 1 and 2 can be identified (S3-guidelines DDG for diabetes in children and adolescents). A position statement by the DDG and other stakeholders is currently being developed to provide further guidance on how to best implement these measures. After a positive IAb result, management and follow-up adhere to international pediatric consensus guidelines.^27^

**Figure 2.**
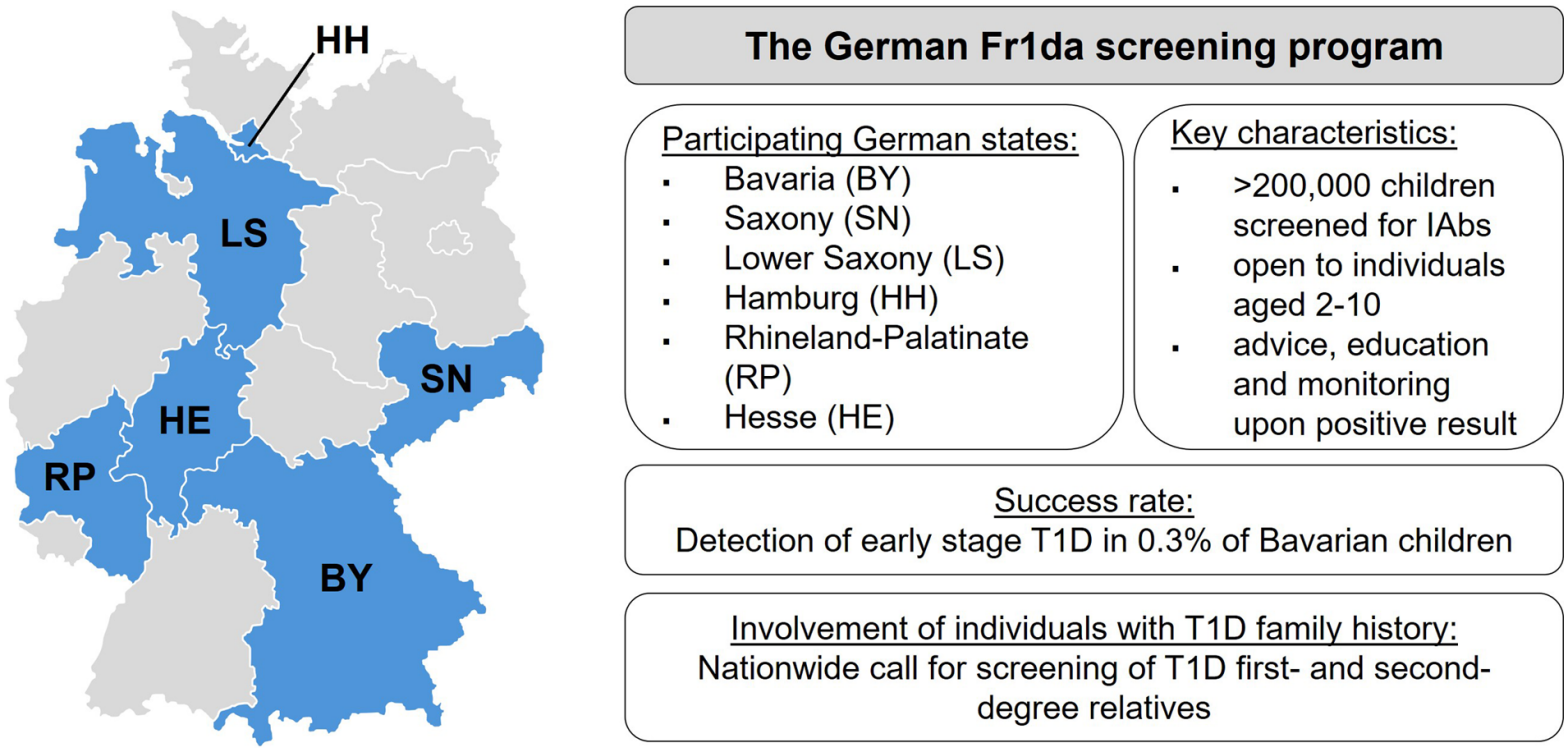
Illustration of the German Fr1da screening program for early detection of individuals with IAbs.

Established in the state of Bavaria, Germany, in 2015, the Fr1da study is the world’s largest open program for early, presymptomatic T1D detection in children, having screened over 200 000 participants (Figure 2). It identified early-stage T1D in 0.3% of Bavarian children^28^ and, since 2022, has been extended free of charge to Lower Saxony, Hamburg, Saxony, Hesse and Rhineland-Palatinate for ages 2 to 10.^22^ Two IAb tests (at ages 2 and 6) achieve 82% sensitivity, 99.8% specificity and a 79% positive predictive value for T1D by age 15.^29^ Multiple IAbs regress in under 1% of cases.^30^ Within the Fr1da study, two screenings in the context of U-checkups (standardized, periodic, pediatric examinations for monitoring a child’s development) are recommended at the age of 3 and 7 to 10 years, respectively. A capillary blood sample is analyzed centrally and, if positive, followed by a confirmatory venous draw. Importantly, the screening invitation has been extended to first- and second-degree relatives on a nationwide basis. If someone has a parent, child, (half-)sister, (half-)brother, aunt, uncle, cousin, niece or nephew with T1D, has not been diagnosed with T1D, and is aged 1 to 21, they are eligible to participate in the screening study by providing a venous blood sample. Once early-stage T1D is confirmed, participants are referred to specialized centers for metabolic staging, comprehensive disease education and, if applicable, psychosocial support.^22^ While this indispensable service is currently financed by research funds, an implementation of a comparable program in Germany’s routine care is not anticipated in the near future.

As these efforts are ongoing in the framework of a controlled study setting, integration of screening members of T1D families into clinical practice and routine care is a next step that is actively discussed for the German T1D landscape, but faces key challenges in organization, ethical considerations, financing and capacity. To achieve this, clear assignment of responsibility is required: pediatricians could collect capillary samples during standard U-checkups, while general practitioners or diabetologists could perform the same procedure for adult patients. In both cases, specialized diabetes prevention centers to be established on a large scale would oversee follow-up for families with T1D history. These centers would not be limited to familial cases but would serve as regional referral hubs for any person identified with positive IAb status or early-stage T1D. Although this adds to the workload of health care professionals (HCPs) who must perform sampling, convey results and provide initial counseling, an evaluation of the Fr1da study found that most of the participating primary care pediatricians and pediatric diabetes care centers supported permanent integration into standard care.^31^ However, since only participating Fr1da providers who completed the evaluation (48% of pediatricians and 56% of centers) were surveyed, these views may not be fully generalizable to the broader provider population. For individuals with a family history of T1D, targeted IAb testing can provide a clearer, individualized estimate of risk compared with a nonspecific familial predisposition, while importantly acknowledging residual uncertainty and the need for counseling and psychosocial support. Samples should then be processed in dedicated screening hubs with interconnected laboratories to ensure quality and efficiency. Scaling will likely demand investment in automation and staff training. In the Fr1da study (Bavaria), the observed cost per child screened was €28.^32^ Transition of the study setting to a standard-care scenario would additionally need to account for medical practice, sample coordination, and clinical/psychosocial follow-up of children with presymptomatic T1D, which will need to be addressed through future reimbursement and staffing models in routine care.

For the German perspective, modeling suggests that population-wide screening could boost the number of children under pediatric T1D care by up to 60% over the next two decades, but long-term savings from fewer DKA events and hospitalizations would more than offset initial expenditure.^33^ By identifying more children, the number of T1D-affected families will inevitably grow. Digital platforms for automated result reporting and for scheduling follow-up consultations to discuss positive findings as well as future AI-driven risk stratification could help alleviate capacity constraints.^34^ Especially individuals with a family history of T1D receiving a positive screening result may benefit from such personalized follow-up strategies that aid integrating future T1D management aspects into daily routines. Establishing a robust, family-focused screening program in Germany would both mitigate DKA risk in high-risk relatives and pave the way for broader population screening efforts.^35^ Indeed, implementation of such a protocol completely prevented DKA at clinical onset in this high-risk cohort in Italy.^36^

In addition to practical challenges, resonance among German experts with regard to a T1D screening in families of elevated risk is mixed. Proponents emphasize earlier intervention, lower DKA risk at onset and better outcomes through closer monitoring and preventive research; critics caution against false positives, psychological strain on families and the financial and logistical burdens of a nationwide family-based rollout.^37^ Although early detection can lead to unnecessary anxiety (nearly 25% of IAb-positive children remain asymptomatic after 10 years),^2^ those from families with a T1D history who screened positive generally experienced lower rates of DKA, better HbA_1c_ and shorter hospital stays at diagnosis.

Yet, after a 5-year follow-up period, these advantages disappear,^38^ raising the question of whether benefits derive from the screening result itself or simply from heightened awareness due to family history which has been shown to be a protective factor against developing DKA at onset in individuals with no prior IAb screening.^39^ Compared with age-matched children diagnosed with diabetes in the community (non-TEDDY cases), TEDDY children (identified within the TEDDY study through testing of at-risk individuals) showed differences in diabetes-specific quality of life and their parents reported lower frequency and difficulty of parenting stress; parent diabetes-specific anxiety did not differ between groups.^40^ Public education alone can have an effect on DKA: the Stuttgart campaign informing parents about T1D symptoms cut DKA at diagnosis from 28 to 16% over 3 years without any IAb testing.^41^ Still, this improved rate of DKA at onset is markedly above the rate observed in the Fr1da study, in which a percentage of 2.5% of DKA at manifestation was evident.^23^

Despite enhanced educational outreach, DKA rates are rising globally.^42^ Even people with T1D often lack familiarity with DKA’s signs and triggers^43^ and their first- and second-degree relatives, though at elevated risk, are similarly ill-prepared to recognize impending symptoms. Prospective studies conducted in people with a T1D family history report DKA rates near 40%, despite the participants’ familiarity with the disease, which could, however, be lowered with prior screening efforts.^44,45^ Given first-degree relatives carry a roughly 15-fold increased risk (twofold for second-degree),^46^ they remain the highest-priority group for targeted screening programs. Any expansion, however, must respect voluntary participation and individuals’ right not to know their risk.

### The International Perspective

On a global scale, dedicated consortia now coordinate early T1D detection in relatives, both to identify at-risk individuals and to recruit them for prevention trials. Since 2004, the US TrialNet Pathway to Prevention Study has screened 220 000 relatives (3% multiple IAb+).^47-49^ Europe’s INNODIA partnership (4,400 relatives, 2.6% multiple IAb+),^47,50^ Colorado’s ASK program (screening for T1D and celiac autoantibodies)^51^ and T1Detect (based in the United States and launched by JDRF) likewise screen at-risk individuals from public and relatives.^52^

Single countries have moreover committed to early T1D detection measures through national initiatives (Table 1). In 2023, Italy became the first country to legislate nationwide screening for T1D and coeliac disease in 1- to 17-year olds, including those with a family history, piloting the implementation in four regions (D1Ce study in Campania, Lombardy, Marche, Sardinia) before full rollout in 2025. Although Italian pediatricians generally support the program, many report gaps in training and resources. Notably, 36% of diabetes centers lack dietitians and 41% lack psychologists to deliver multidisciplinary follow-up care.^53,54^ Consequently, guidance for monitoring has been proposed for children that are identified with early-stage 1 T1D in Italy.^55^ France, by contrast, issues a position statement recommending at least one IAb test for relatives of people with T1D up to age 45, with the possibility of repeated testing at least once after a negative result.^56^ In the United Kingdom, clinicians are debating a national approach that would combine GRSs with IAb screening, despite capacity constraints.^57,58^

**Table 1.** Overview of International Screening Consortia and Selected National Approaches Toward Early T1D Detection in At-Risk Individuals.

Selected national approaches toward early T1D detection in at-risk individuals			
Country	Recommended screening population	Recommended screening age	Further information
**Italy**	Everyone in the recommended age span	1-17	Nationwide effort; also includes screening for coeliac disease
**France**	Relatives of people with T1D	Up to the age of 45	Negative screens should be repeated every 4 years until age 12
**United Kingdom**	Nationwide screening programs discussed	Aligning screening with routine child health contacts discussed	Exploring options of both IAb screening alone and in combination with GRS
**United States**	Relatives of people with T1D	Regardless of age	Introduction of new ICD-10 classifications
Worldwide screening consortia			
Name of the consortium	Number of individuals screened	Rates of positive screens (≥2 IAbs)	
**TrialNet**	>250 000	2.5%	
**INNODIA**	>4 000	2.6%	

Source: Numbers for international efforts have been taken from Sims et al.^47^

As early-stage T1D before onset of hyperglycemia is increasingly recognized as a distinct autoimmune disease stage, the latest release of the International Classification of Diseases (ICD-10) codes by United States-based Center for Disease Control also appreciates the importance of an early diagnosis of presymptomatic T1D, alleviating insurance and reimbursement issues as well as allowing for standardization and epidemiological tracking. Effective October 2024, the new release includes codes for E10.A0—T1D mellitus, presymptomatic, unspecified; E10.A1—T1D mellitus, presymptomatic, Stage 1; E10.A2—T1D mellitus, presymptomatic, Stage 2.^59^ Meanwhile, the American Diabetes Association (ADA) has formally endorsed family history-based IAb screening since 2014 in a setting of clinical studies,^60^ only before expanding this recommendation to screening outside of research settings in 2022.^61,62^ In a recent round table statement on T1D screening and awareness organized by the ADA, experts endorsed a stratified public-health strategy beginning with those at highest risk (children and individuals with a family history). Rigorous documentation of family history in electronic health records, scanned for risk factors, could streamline outreach.^63^ Collectively, these national and international initiatives, bolstered by updated diagnostic frameworks, underscore the clinical and economic value of focused early-detection strategies in at-risk families of people with T1D.

## Practical Applications and Follow-Up Strategies on Positive IAb-Screening Results

### Definition of a Screening Algorithm

Early detection of islet autoimmunity is crucial to potentially delay T1D onset, especially in individuals with family history. Autoimmune progression varies, and a robust screening and follow-up strategy can improve longevity, reduce morbidity, and ease family burden.^47^ Current evidence recommends starting IAb screening by age 2 (or even by age 1 for relatives as in Fr1da) despite rare toddlers seroconverting earlier and progressing rapidly to DKA.^59,64^ To balance detection sensitivity and psychological impact of screening, individuals at high risk like first-degree relatives might benefit from integration of T1D GRS, family history assessment, and serial IAb measurements. This combined approach enabled T1D prediction more precisely in contrast to IAb testing alone.^65^ For relatives of people with T1D, a negative pediatric screen warrants one repetition in testing until age of 18, whereas a single negative adult screen may suffice to exclude the development of T1D prestages.^59,66^ A single IAb positivity in childhood calls for a nuanced approach: if seroconversion reverses within 2 years, the 15-year progression risk is 12%; if it persists, risk rises to 30%.^67^ Although less likely, single positivity for one IAb can lead to onset of T1D in children.^2^ Two or more IAbs define early-stage T1D which can be associated with varying progression rates, based on type of detected IAb and age of seroconversion.^68-70^ However, individual progression rates may vary, necessitating monitoring strategies. Clinically, the main IAb targets include IAA, GADA, IA2A, and ZnT8A, and a variety of assay methods are available for their detection.^47,66^

### Involvement of Health Care Providers—Practical Guidance in Managing Positive Screening Results and Pathway for Referral

**Figure 3.**
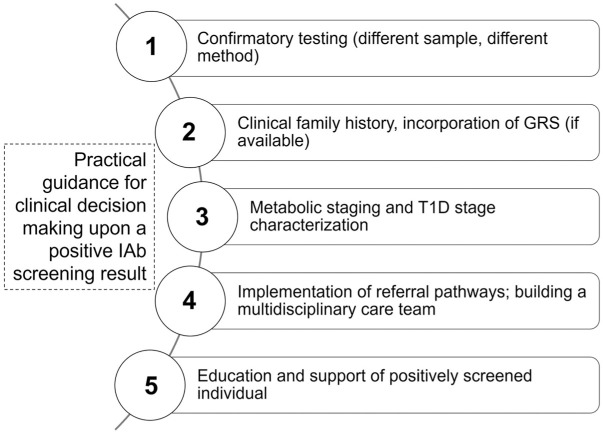
Recommendations for health care professionals on managing patients with a positive islet autoantibody screening result. Figure based on Phillip et al,^66^ Haller et al^72^ and American Diabetes Association Professional Practice Committee.^73^

In clinical practice, a streamlined care plan for individuals identified as IAb-positive (IAb^+^) must address the ethical, logistical and financial hurdles of family-based screening, requiring coordinated efforts among policymakers, health care providers and funders as well as unified national guidelines to align with international recommendations.^35,71^ Germany, lacking a national recommendation, relies on study-based regional programs for the general population (Fr1da) as well as nationwide options for individuals with a T1D family history (Fr1da for relatives). As of now in the Fr1da study, a positive result in family-based IAb screening warrants referral to specialized centers or pediatric diabetologists. Upon a positive autoantibody result, the primary care provider or pediatrician confirms and documents IAb^+^ status, coordinates follow-up metabolic monitoring and facilitates timely referral to a pediatric endocrinologist as progression risk increases. Clear communication (updating electronic records and sharing status with the care team consisting of primary care providers, pediatricians, diabetologists, endocrinologists and nurses) ensures continuity. Pediatric endocrinologists and diabetologists then individualize follow-up by choosing between oral glucose tolerance test (OGTT), continuous glucose monitoring (CGM) or self-monitoring of blood glucose (SMBG) based on age, antibody profile and glycemic trends. Continued education for primary HCPs is encouraged,^66^ as T1D staging criteria are relatively new. Psychosocial support may be embedded in routine visits through brief emotional screenings, with referral to specialized pediatric psychologists if moderate to severe distress emerges. Escalation to specialist care occurs when dysglycaemia criteria are met (persistent hyperglycemia with or without symptoms). At this point, pediatric endocrinologists assume primary oversight and intensify psychological support and insulin treatment may be initiated as appropriate. In any case, follow-up after a positive test should involve HCPs who are familiar with the significance of the screening result.^47^ Also, HCPs must be aware that IAb-positive individuals exhibit varying progression rates, with some developing symptoms rapidly and others remaining unaffected for decades. These recommendations draw on consensus guidelines,^66,72^ but German stakeholders are required to translate those published standards into concrete, nationally standardized workflows by defining clear responsibilities, processes and resources. Clinical decision-making can follow the steps proposed in Figure 3.^66^,^72,73^

## From Concept to Practice: Aspects of the Health Care Delivery Task

### Monitoring Procedure in IAb-Positive Individuals With Early-Stage T1D

In IAb^+^ individuals, precise metabolic staging of the presymptomatic T1D should follow to assess remaining beta cell function and the necessity for initiation of insulin treatment,^66,72^ typically after referral to a specialist. As monitoring methodology, CGM, OGTT and HbA_1c_ measurement are most suitable. A comprehensive overview of different methods is given in Table 2. While OGTT may not always be feasible or acceptable, especially in children, alternative approaches include fasting plasma glucose (FPG) assessment, intermediate OGTT timepoints (CGM may emerge as an alternative),^74^ and 2h plasma glucose following oral glucose load.^72^

**Table 2.** Monitoring methodologies of early-stage T1D, along with individual strengths and weaknesses as well as practical considerations for their application. Modified after a recent monitoring consensus statement.^66^ Practicable procedures still need to be defined and consented for Germany.

Method	Purpose	Frequency	Main strengths/weaknesses	Practical guidance
Standard OGTT (2h)	Diagnose T1D & monitor progression	Depending on age and stage	+ Gold standard for glucose intolerance detection - Time-consuming, medium adherence, requires fasting	● Adults: Standard 75 g glucose load after 8 h fast (adaption for children: 1.75 g glucose per kg bodyweight, maximum of 75 g) ● Draw at 0/120 min (30/60/90 min optional) ● Use ADA criteria for interpretation^108^
Random glucose	Acute hyperglycemia detection	As needed	+ Quick, no fasting required - Low specificity for early-stage T1D	● Interpret >200 mg/dL with symptoms as diagnostic ● Always confirm with OGTT
Standard HbA1_c_ test	Long-term glycemic control	Every 3-6 months	+ Reflects 3-month average, no fasting - Insensitive for early dysglycemia	● Avoid in anemia/ long-term kidney disease^109^ ● Consider correlation with corrected fructosamine if unreliable^110^
CGM	Continuous glucose patterns	Continuous use	+ Reveals glycemic variability & trends - Lower predictive value than OGTT metrics	● Intermittent use of professional CGM for early stages^111^ ● Focus on time-in-tight range (70-140 mg/dL)^66^
SMBG (self-monitoring of blood glucose)	Daily glucose monitoring	Multiple daily	+ Immediate feedback, guides management - Invasive, adherence challenges	● 4-point profiles (fasting/pre-prandial/2h postprandial/postabsorptive) ● Calibrate meters every 6 months
C-peptide	Beta-cell function assessment	With OGTTs	+ Quantifies insulin production, stages disease - Requires standardized testing conditions	● Measure fasting + stimulated (post-OGTT) C-peptide, providing both metabolic and functional data ● Use ≥0.2 nmol/L as preservation threshold
Repeat IAb testing	Autoimmunity monitoring	Up to once, depending on IAb status, age and family history	+ Tracks immunological progression - Doesn’t directly assess metabolic status	● Repeated testing for IAA, GADA, IA2A and ZnT8A is indicated ● ZnT8A titer determination predictive in high-risk individuals such as individuals with family history^112^
Education	Risk communication and adherence	Ongoing	+ Improves OGTT compliance when effective, explains individual risk of progression - Quality of education impacts outcomes, cultural variations	● Numerical risk communication (e.g., 70/100 risk) for clear understanding^113^ ● Use material specifically developed for educating individuals with early-stage T1D^22^

In individuals with family history, T1D progression mirrors that of the general population and realtime measures like CGM and SMBG help prevent DKA.^75,76^ Recent consensus guidelines outline monitoring strategies:^66^ personalized CGM metrics can identify children at imminent risk,^77^ while adults with multiple IAbs may undergo annual metabolic checks in primary care. If normoglycemia persists for more than five years in stage 1, a monitoring visit every second year is indicated; progression to stage 2 warrants biannual monitoring including HbA_1c_ and an additional method.^66^ Although seroconversion peaks before age 1,^26^ over half of T1D cases arise in adults,^78^ highlighting the need for a multidisciplinary approach in individuals above age 18, especially when a T1D family history is present.^79^

### Therapeutic Strategies for Modulation of Early T1D Disease Course

Detecting T1D early enables intervention strategies aiming to prolong insulin independence and improve glycemic control and long-term outcomes in high-risk individuals, particularly those with a family history of T1D. Historically, clinical studies have enrolled predominantly first- and second-degree relatives of T1D patients, given that these remain prime candidates for immunomodulatory DMT treatment and offer pragmatic advantages, namely an increased genetic predisposition and heightened disease awareness within these familial cohorts.^80^ A key class of immune modulators are anti-CD3 monoclonal antibodies blocking lymphocyte activation.^81^ Teplizumab, a humanized anti-CD3 antibody, can induce partial T-cell exhaustion leading to diminished autoreactivity to preserve residual beta cell function.^82^ In a placebo-controlled trial of stage 2 T1D patients (with a family history), a single 14-day course delayed progression to clinical T1D by a median of 2.7 years, with 50% of teplizumab-treated but only 22% of the placebo-treated remained diabetes free over a median follow-up time of 2.5 years.^83,84^ Teplizumab received Food and Drug Administration (FDA) approval in November 2022 for delaying stage 2 to 3 T1D in individuals aged 8 years and above.^85^

Besides anti-CD3 DMTs, several other immunomodulators are under clinical investigation, primarily in stage 3 T1D. T cell co-stimulation blockade with abatacept (CTLA-4-immunoglobulin) preserved C-peptide after two years in new-onset T1D but showed no benefit in stage 1 relatives after one year.^80,86^ Anti-thymocyte globulins (ATGs) depleting thymocytes (early stage of T cells) maintained C-peptide secretion and reduced HbA_1c_ in individuals with stage 3 T1D.^87,88^ Alefacept (LFA-3-Immuboglobulin targeting CD2) halved hypoglycemia episodes and lowered insulin needs over 24 months, with increased meal-stimulated C-peptide but without changing overall glycemic control.^89^ Beyond T cell targets, B cell depletion with rituximab, cytokine blockade (ustekinumab against IL-12/IL-13), JAK-STAT inhibition (baricitinib), tumor necrosis factor (TNF)-α blockade (golimumab), and beta-cell protection with verapamil are all in various phases of trials.^90-94^ Combination strategies, such as IL-21 inhibition plus the glucagon-like peptide-1 (GLP-1) receptor agonist liraglutide, are also being explored.^95^ These agents vary in potency and durability and ongoing studies aim to refine sequential or combinatorial regimens to more effectively delay T1D progression.

Although a T1D family history aids early risk stratification, it does not confer differential DMT efficacy,^35^ and these therapies require careful safety surveillance for infections, malignancies, and cytopenias,^96^ with tailored monitoring protocols indicated.^97^ Current DMT regimens only delay, rather than prevent, clinical onset, indicating a need for future continuous or combinatorial strategies guided by novel biomarkers to personalize intervention.^98^

## Emerging Health Care Directions for Future Management of Individuals With a T1D Family History in Germany

### Research and Policy Implications

Over the past decade, initiatives like Germany’s Fr1da screening have enabled presymptomatic T1D diagnoses and led to hundreds of children being identified before stage 3 onset. Family history was more frequent than in studies that did not conduct IAb screening prior to manifestation, underscoring the potential such screening initiatives hold particularly for relatives of individuals with T1D.^23^ However, despite consensus guidelines on early-stage monitoring,^66,72^ individualized assessment prevails, connected to the inherent uncertainty of progression speed to an advanced T1D stage. This leaves a significant gap, especially among adults with prestage T1D, where data on the monitoring period is scarce and unmet medical needs for adequate follow-up persist.^66^ The Global Platform for the Prevention of Autoimmune Diabetes (GPPAD) preventive trials use general-population newborn screening to identify infants at elevated genetic risk for islet autoimmunity, regardless of family history. However, progression in older children and adults remains underexplored. The UK’s T1DRA study will screen 20 000 adults (18-70 years) to establish monitoring protocols for IAb-positive individuals and support immunotherapy trials.^99^ Ongoing psychological support is also needed in families with T1D, especially when both parent and child have T1D, which can strain caregivers’ own health management.^100^ Finally, equitable access to and reimbursement for IAb screening, extended monitoring and a broader array of DMTs are essential to ensure comprehensive prevention and care for all at risk.

### Next Steps for German T1D Prevention Efforts

Over the coming years, Germany must bolster early T1D detection and prevention to address a projected 18% to 32% rise in incidence by 2040 (translating to roughly 80 000 new cases).^101^ Despite stable German DKA rates at onset over the last 20 years, numbers remain higher than in Finland or Sweden.^102,103^ Integrating polygenic GRS into newborn screening, especially for those with a family history, could identify high-risk infants before disease initiation.^104^ The 2025 introduction of the opt-out electronic patient record (ePA) may enable clinicians to identify T1D risk factors such as familial predisposition, track IAb status and glycemic trends centrally and coordinate care across specialists.^105^ Germany should also adopt ICD-10 presymptomatic T1D codes which are currently absent from the annually updated ICD-10-GM (German modification) to standardize national T1D staging and documentation.^106^ Finally, research must pivot toward matching individuals to emerging DMTs and evaluating immunotherapy safety and efficacy.

## Conclusion

Lessons and solutions for German T1D efforts should be incorporated into a “white paper” or consensus statement describing evidence-based insights from a clinical, economic and educational perspective. Current evidence underscores the dual imperative of refining family history-based screening for T1D while expanding population-wide approaches. In Germany, screening programs (e.g. Fr1da) have successfully identified high-risk children, yet approximately 90% of T1D cases arise outside familial contexts.^20^ In Finland, extending family history analyses cut nonfamilial case incidence to under 80% and enabled earlier diagnosis through greater symptom awareness,^46^ highlighting the impact of systematic prevention strategies that Germany could similarly harness, particularly by encouraging at-risk relatives to participate in the German Fr1da study program for relatives. As novel DMTs reshape the T1D treatment landscape, standardized post-screening monitoring protocols will be essential to optimize therapeutic windows and reduce DKA at clinical onset. Ensuring equitable access, particularly between rural and urban areas, to both screening and emerging treatments is equally critical. By combining familial risk stratification with population-level screening and rapid DMT integration, Germany can mitigate projected increases in T1D incidence and severity. Stronger research-care partnerships, continued DMT development, deeper insight into T1D heterogeneity and expanded screening initiatives^107^ will collectively drive precision-focused prevention.
